# Alpha4 Na,K-ATPase Localization and Expression Are Dynamic Aspects of Spermatogenesis and in Sperm Incubated Under Capacitating Conditions

**DOI:** 10.3390/ijms26051817

**Published:** 2025-02-20

**Authors:** David Milewski, Paul F. James

**Affiliations:** Department of Biology, Miami University, Oxford, OH 45056, USA; david.milewski@nih.gov

**Keywords:** Na,K-ATPase alpha4, ATP1A4, spermatozoa, Sertoli cell, testis, capacitation

## Abstract

Utilizing high-resolution microscopy in conjunction with a new antibody highly specific for rat alpha4 Na,K-ATPase, we describe changes in alpha4 expression during spermatogenesis and in sperm incubated under capacitating and noncapacitating conditions. Immunohistochemical analyses showed alpha4 expression at low levels in spermatogonia and in pachytene spermatocytes. Alpha4 then becomes highly expressed on round spermatids and the midpiece of elongated spermatozoa within the seminiferous tubules. In noncapacitating conditions, alpha4 was confined mainly to the flagellum of mature sperm; however, under capacitating conditions, sperm acquired intense alpha4 staining along the acrosomal region of the sperm head. To visualize the precise localization of alpha4 in the sperm head, we performed an ultrastructural analysis using immuno-scanning electron microscopy. Under capacitating conditions, sperm exhibited alpha4 staining along the dorsal surface of the sperm head associated with the acrosome. In addition, after 4 h of incubation in motility buffer, we observed an increase in alpha4 protein in sperm that could be blocked with chloramphenicol, a mitochondrial-type ribosome inhibitor. These findings demonstrate that both the localization and expression level of alpha4 Na,K-ATPase are dynamic aspects of sperm maturation and suggest that sperm motility and capacitation may be supported by these changes to the location and amount of this protein.

## 1. Introduction

Na,K-ATPase is an electrogenic integral membrane protein which hydrolyzes ATP to actively transport Na^+^ out of the cell and K^+^ into the cell [1]. The cell relies on this electrochemical gradient to maintain fundamental cell physiological properties such as membrane potential, osmolarity, and intracellular pH. Functional Na,K-ATPase is minimally a heteromeric protein complex composed of a catalytic alpha subunit which hydrolyzes ATP and binds cations and a beta subunit required for plasma membrane localization [1]. Of the four Na,K-ATPase alpha subunit isoforms described to date (alpha1, alpha2, alpha3, alpha4), male germ cells express the alpha1 and alpha4 isoforms. Alpha1 Na,K-ATPase is ubiquitously expressed in mammalian tissues and is therefore thought to play a role in maintaining basal ion gradients. The alpha4 isoform, however, has been reported to be expressed exclusively in male germ cells, suggesting a tissue-specific function [2,3,4,5].

Although they are structurally very similar, the alpha1 and alpha4 isoforms each possess unique biochemical properties. Compared to the alpha1 isoform, alpha4 has a higher affinity for Na^+^, a lower affinity for K^+^, and an equivalent affinity for ATP [6,7]. In addition, alpha4 has a higher affinity for the inhibitor cardiac glycoside ouabain compared to the alpha1 isoform, allowing for the selective inhibition of the alpha4 isoform in the presence of the alpha1 isoform [3,5,7].

Pharmacological inhibition of the alpha4 Na,K-ATPase isoform alone is sufficient to inhibit sperm motility, whereas inhibition of both alpha1 and alpha4 together induces no further reduction in motility, suggesting that, of the two Na,K-ATPase alpha isoforms expressed in sperm, the alpha4 isoform is a specific regulator of sperm motility [7,8]. In agreement with this, male alpha4 Na,K-ATPase-null mice are completely sterile due to immotile sperm [9,10]. The loss of motility following disruption of alpha4 Na,K-ATPase activity coincides with reduced membrane potential, increased [Ca^2+^]i, and, likely, reduced Na/H exchanger activity, leading to acidification of the sperm [11,12]. In addition, alpha4 Na,K-ATPase-null mice sperm from the caudal epididymis display structural defects resulting in a bend in the flagellum at the junction of the midpiece and principal piece [13], and alpha4 Na,K-ATPase knockout sperm display energetic defects including reduced glycolytic flux and impaired mitochondrial function [14]. Altering the affinity of the two sperm Na,K-ATPase isoforms (alpha1 and alpha4) to a Na,K-ATPase inhibitor in genetically modified mice confirmed that Na,K-ATPase alpha4 is the Na,K-ATPase necessary for the majority of sperm motility [15]. Taken together, these data demonstrate the critical importance of the Na,K-ATPase alpha4 specifically in the physiology of sperm.

Immunocytochemistry has shown that alpha4 is localized to the flagellum of human and rodent sperm [3,4,8], a localization that supports its essential functional role in sperm motility. However, in addition to supporting basic motility, studies suggest that the alpha4 Na,K-ATPase may also be involved in the process of sperm capacitation. Sperm capacitation is associated with multiple biochemical events, which renders the sperm competent for fertilization. The sequence and requirement for these events varies between organisms, but in general, capacitation involves the alkalinization of the sperm cytoplasm [16], influx of calcium and bicarbonate ions [17,18,19], increase in plasma membrane fluidity by cholesterol efflux [20], and the cAMP-dependent activation of protein kinases, which results in the rapid and widespread phosphorylation of tyrosine residues of target proteins [21]. These biochemical events encompass two physiological transitions within the cell. First, sperm attain hyperactive motility, which is associated with increased beat frequency and linear velocity. This increase in motility likely helps sperm navigate through the female reproductive tract and penetrate the zonae pellucidae. Second, capacitation prepares the sperm to undergo the acrosome reaction.

In rat, mouse, and human sperm, the alpha4 Na,K-ATPase has been reported to be expressed primarily in the flagellum [3,4,8], and a VxP motif has been identified in the human alpha4 Na,K-ATPase that has been proposed to be important to its flagellar localization [22]. In bovine sperm, alpha4 localizes to the flagellum and has been reported to also be found in the sperm head [23]. Interestingly, the bovine sperm Na,K-ATPase alpha4 isoform has been reported to redistribute during capacitation, moving from a diffuse distribution across the sperm head to a restricted post-acrosomal localization [23]. In addition, the Na,K-ATPase alpha4 isoform has been shown to undergo a shift in both localization and activity in rat sperm during capacitation; as capacitation progresses, the level of alpha4 protein found on the plasma membrane rises, and its activity in sperm increases [24]. The bovine Na,K-ATPase has been found to interact with unique and overlapping sets of proteins in both lipid raft and non-raft fractions of capacitated sperm [25,26]. These alpha4 Na,K-ATPase interacting proteins include signaling molecules, receptors, kinases, proteases, and adaptor proteins, suggesting a role for alpha4 in bovine sperm capacitation and hinting at potential mechanisms for its redistribution [25,26,27].

It is not clear what role alpha4 plays in capacitation, as varied effects of Na,K-ATPase inhibition on capacitation have been reported for different species. Na,K-ATPase activity has been reported to be necessary for the acrosome reaction in the hamster [28], whereas in bovine sperm, Na,K-ATPase inhibition increased the proportion of sperm undergoing lysophosphatidylcholine-induced acrosome reactions [29] as well as induced tyrosine phosphorylation and spontaneous acrosome reactions [23]. In mice, inhibition of Na,K-ATPase activity increased the rate of capacitation but did not increase the number of acrosome-reacted sperm [30], and it was shown that specific inhibition of Na,K-ATPase alpha4 activity prevents rat sperm from acquiring hyperactive motility and partially prevents membrane hyperpolarization, but does not alter the incidence of spontaneous acrosome reaction [24]. Nonetheless, the reports of the involvement of the Na,K-ATPase activity in capacitation-associated events across multiple species, taken together with the reports of specific changes in alpha4 activity and localization during capacitation, suggest that alpha4 Na,K-ATPase may be an important component of capacitation and/or the acrosome reaction.

Alpha4 Na,K-ATPase has a well-established role in maintaining motility in rodent, human, and bovine sperm [8,9,10,12,29]. However, the function of alpha4 may depend on the specific stage of sperm maturation both within the seminiferous tubule as well as in the female reproductive tract. Since sperm possess functionally distinct regions, we have performed a detailed analysis of the expression and localization of alpha4 Na,K-ATPase. Here, we report the first ultrastructural localization of this testis-specific isoform and demonstrate that alpha4 appears on the plasma membrane of the sperm acrosomal region during capacitation. In addition, we demonstrate the presence of alpha4 Na,K-ATPase in what appear to be phagosomes in Sertoli cells and provide evidence supporting an increase in the total alpha4 Na,K-ATPase content in sperm during incubation under both noncapacitating and capacitating conditions.

## 2. Results

### 2.1. Generation of a New Anti-alpha4 Polyclonal Antibody

**Figure 1:**
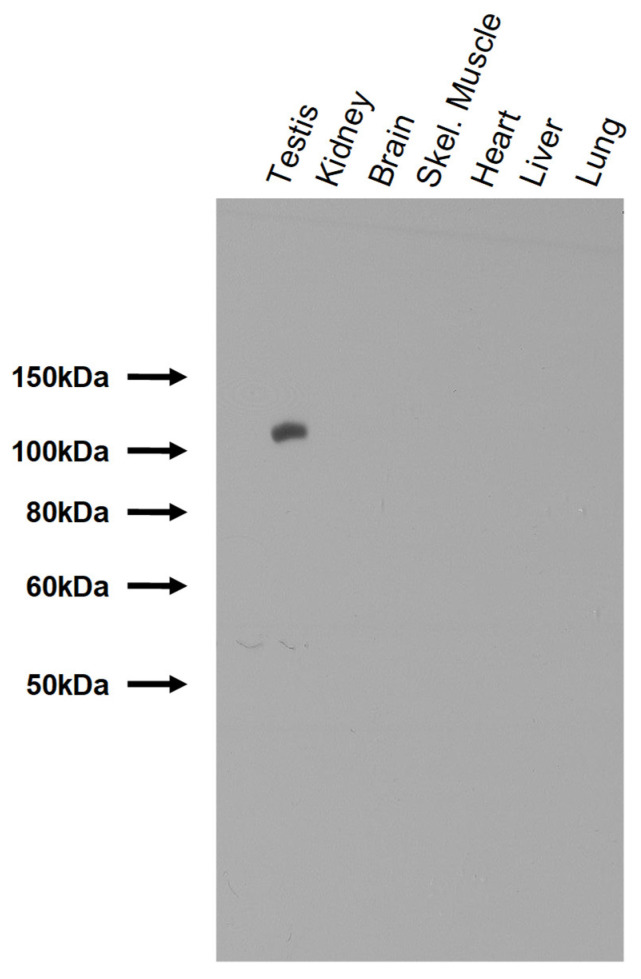
Characterization of a new polyclonal anti-alpha4 Na,K-ATPase antibody. Total protein was extracted from various rat tissues and separated by SDS-PAGE. The blot was probed with our newly generated anti-alpha4 antibody, labeled with an HRP-conjugated goat anti-rabbit secondary antibody, and exposed on autoradiographic film. A single, testis-specific band was produced corresponding to the alpha4 Na,K-ATPase.

Given the importance of catalytic alpha4 Na,K-ATPase activity in regulating motility, we have sought to address the possibility that alpha4 Na,K-ATPase localization and expression is a spatially and temporally dynamic aspect of sperm maturation. To test this, we have generated a sensitive, highly specific polyclonal antiserum against amino acids 16-33 of rat alpha4 Na,K-ATPase. To characterize the specificity of this antiserum, we probed a multi-tissue Western blot with our anti-alpha4 Na,K-ATPase antibody. We observed a single, testis-specific band of approximately 110 kDa corresponding to the predicted size and tissue distribution of the alpha4 Na,K-ATPase polypeptide (Figure 1). This demonstrates that our antibody is highly specific for the alpha4 Na,K-ATPase polypeptide.

### 2.2. Alpha4 Na,K-ATPase Is Found in Spermatogenic and Presumptive Sertoli Cells of the Testes

**Figure 2:**
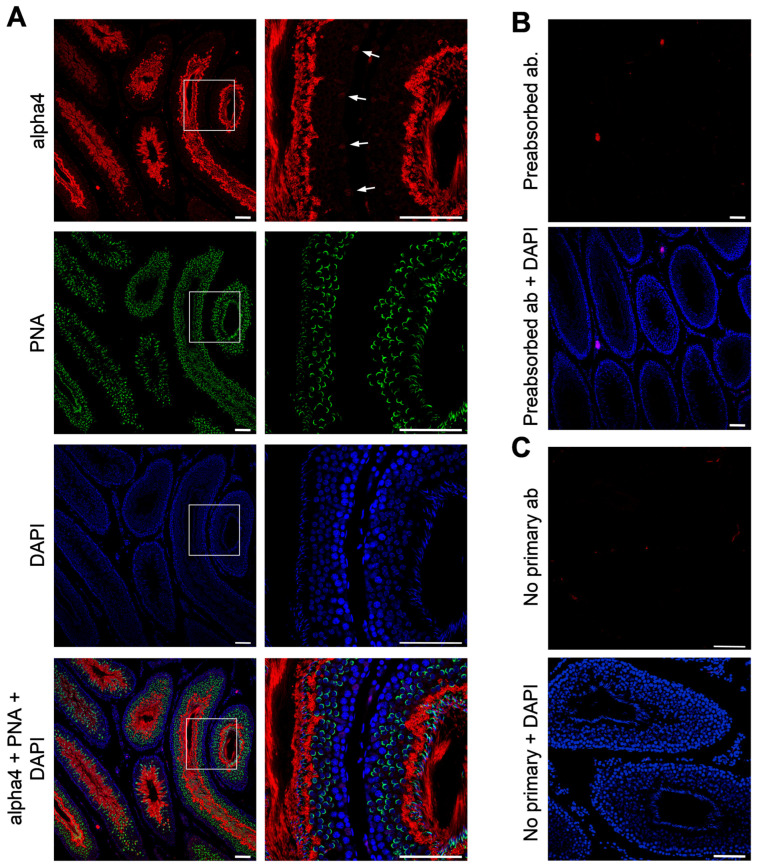
Immunohistochemistry of alpha4 Na,K-ATPase and the acrosomal compartment during spermatogenesis. Paraffin sections of rat testis were probed for alpha4 and the acrosomal compartment using anti-alpha4 primary/Alexa 594 secondary antibodies and PNA-FITC, respectively. All sections were counterstained with DAPI. (**A**) Low (left) and high magnification (right) images for alpha4 expression and acrosome staining in the seminiferous tubules. The arrows in the top right panel in (**A**) indicate alpha4 staining in presumptive Sertoli cells. For controls, (**B**) the anti-alpha4 antibody was preabsorbed with the immunizing alpha4 peptide and subsequently used for immunohistochemistry, or (**C**) the primary antibody was omitted entirely. Mag. bar = 50 µm.

To investigate the synthesis and localization of alpha4 protein in the testis, we performed immunohistochemistry on rat testis sections (Figure 2A). At the very periphery of the seminiferous tubule, expression of the alpha4 Na,K-ATPase protein appears at low levels, with a punctate distribution, in a small subset of cells (indicated by white arrowheads in Figure 2A). Interestingly, alpha4 staining completely overlaps with DAPI staining in these cells, suggesting that alpha4 Na,K-ATPase is limited to the nuclear region of these cells. Based on the location of these cells in the seminiferous tubule, their spacing, and the structure of the nucleus, this alpha4 staining appears to be perinuclear in Sertoli cells. More luminally, modest expression of alpha4 Na,K-ATPase is found in primary and secondary spermatocytes as the cells begin to polarize and develop the primordial acrosome, as seen with PNA staining: this alpha4 expression is diffuse and is excluded from the nuclear region of these cells. Once these spermatogenic cells develop into round spermatids, expression of alpha4 Na,K-ATPase significantly increases and is found exclusively along the plasma membrane. Within the lumen of the seminiferous tubule, alpha4 Na,K-ATPase is found along the surface of elongated spermatozoa, apparently limited to the midpiece of the flagellum. Preabsorbing the anti-alpha4 Na,K-ATPase antibody with the antigenic peptide eliminated labeling within the seminiferous tubules, demonstrating that our affinity-purified antisera is specific for the Na,K-ATPase alpha4 isoform (Figure 2B). Immunofluorescent localization in the presence of secondary antibody alone or the preabsorbed primary antibody with the secondary antibody revealed minor cross-reactivity of the secondary antibody outside of the seminiferous tubules, possibly with Leydig cells (Figure 2B,C). Overall, these results demonstrate that alpha4 Na,K-ATPase is found at low levels in Sertoli cells and at increasing levels in spermatogenic cells as they mature, becoming highly expressed along the plasma membrane of round spermatids and then concentrated in the midpiece of spermatozoa.

### 2.3. Alpha4 Na,K-ATPase Localization Is Dynamic During Capacitation of Epididymal Sperm

Armed with our newly generated, high-affinity anti-alpha4 Na,K-ATPase antiserum, we next sought to examine whether alpha4 Na,K-ATPase was expressed in the head of rat sperm and to explore the possibility that the expression of this protein changes, with respect to levels or location, when sperm are incubated under capacitating conditions.

**Figure 3:**
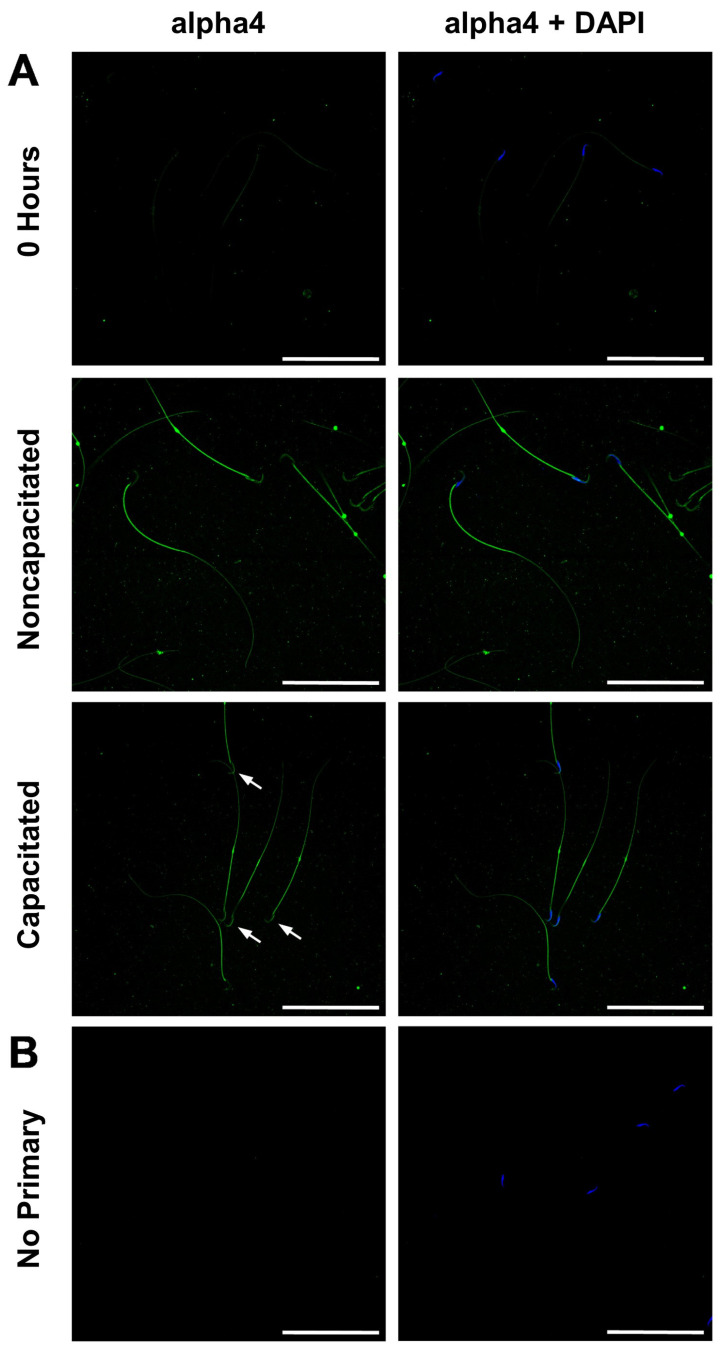
Immunocytochemical localization of the alpha4 Na,K-ATPase in noncapacitated and capacitated sperm. Sperm were isolated from rat caudal epididymes, fixed, and probed for alpha4 (**A**) immediately after isolation (top row) or after being incubated for 4 h in noncapacitating (middle row) or capacitating media (bottom row). The white arrowheads in (**A**) (bottom panel) indicate alpha4 staining in the acrosomal region of the sperm head. (**B**) The primary antibody was omitted to determine the specificity of our secondary antibody. Mag. bar = 20 µm.

To determine where the alpha4 Na,K-ATPase protein is localized in epididymal sperm and if the localization of alpha4 is dependent on capacitating conditions, we isolated fresh sperm from rat caudal epididymes and examined alpha4 Na,K-ATPase localization immediately or after a 4 h incubation in noncapacitating or capacitating mTALP motility buffer. Immediately after isolation from the caudal epididymis, sperm express alpha4 Na,K-ATPase predominantly in the sperm tail, particularly in the midpiece (Figure 3A,B), which is consistent with previous reports on rat sperm [5,11]. However, using our antisera, low levels of diffuse alpha4 Na,K-ATPase staining were also observed in the principal piece and the sperm head (Figure 3A, top two panels). After incubation for 4 h in noncapacitating buffer, an overall increase in alpha4 Na,K-ATPase staining intensity was observed in the sperm flagellum and head (Figure 2A, middle panel). After 4 h in capacitating buffer, sperm displayed an increase in flagellar alpha4 Na,K-ATPase expression similar to that seen in noncapacitating buffer. In addition, we found that capacitated sperm contain an intense region of alpha4 Na,K-ATPase protein, specifically in the acrosomal region of the sperm head (indicated by white arrowheads in Figure 3A, bottom panel). To the best of our knowledge, this is the first account of significant alpha4 Na,K-ATPase expression in the head of rodent sperm, and this capacitation-dependent localization suggests that alpha4 Na,K-ATPase may be playing a role in priming or initiating the acrosome reaction.

### 2.4. Ultrastructural Localization of alpha4 Na,K-ATPase in the Sperm Head

**Figure 4:**
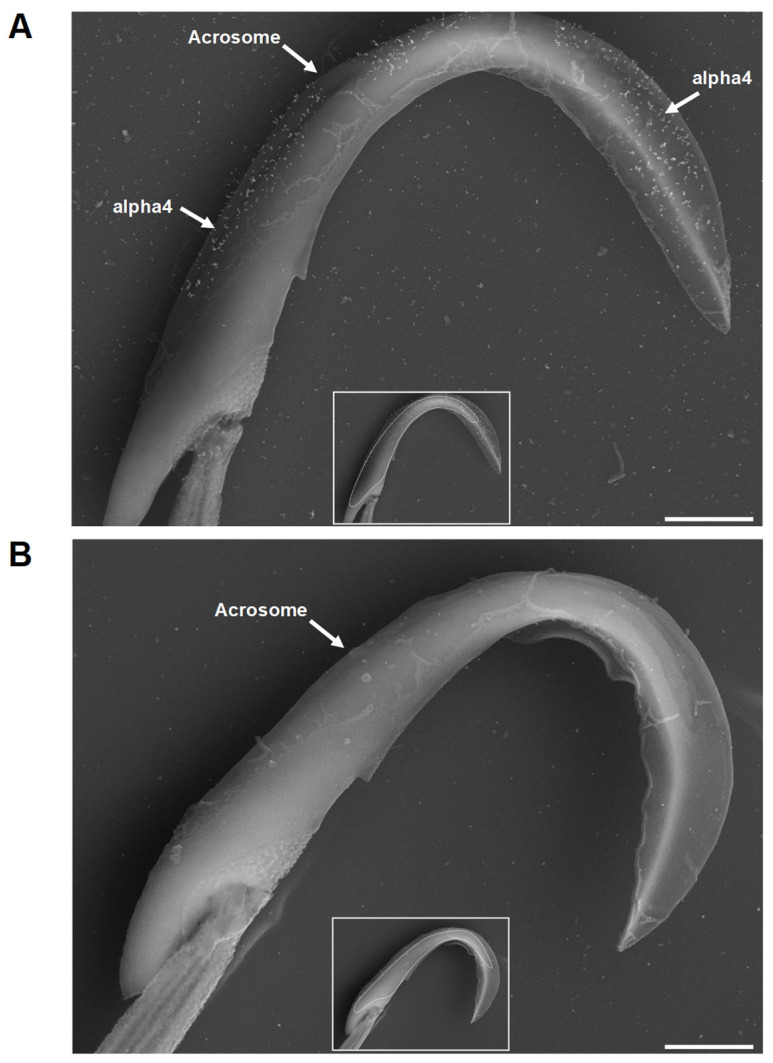
Ultrastructural localization of the alpha4 Na,K-ATPase in the sperm head. Sperm were fixed, mounted onto glass coverslips, incubated (**A**) with or (**B**) without the anti-alpha4 antibody, and labeled with 18 nm colloid gold. Following immunolabeling, sperm were postfixed, dehydrated, carbon coated, and visualized in a scanning electron microscope. Sperm were imaged using a Zeiss Supra 35 VP FEG SEM using a 12 kV accelerating voltage, 7 mm working distance, and 30 µm NA. Nuclei are outlined in the inset images. Mag. bar = 1 µm.

Next, we wanted to more precisely define this isoform’s distribution in the head of capacitated sperm by using high-resolution electron microscopy. Immunogold labeling of alpha4 Na,K-ATPase confirmed that the alpha4 isoform is localized to the head of the sperm (Figure 4). In the head of sperm incubated under capacitating conditions, alpha4 Na,K-ATPase expression was restricted to the acrosomal region, with no alpha4 labeling observed in the postacrosomal region. The presence of the acrosome was indirectly confirmed by visualizing the well-defined nucleus (Figure 4A,B insets) and the lighter acrosomal compartment surrounding it. Interestingly, immunogold labeling of alpha4 Na,K-ATPase was observed only on the sperm plasma membrane; alpha4 Na,K-ATPase protein appeared to be completely absent from the acrosomal matrix and the inner acrosomal membrane.

### 2.5. Na,K-ATPase alpha4 Protein Levels Increase During Incubation of Sperm in Noncapacitating and Capacitating Buffers

**Figure 5:**
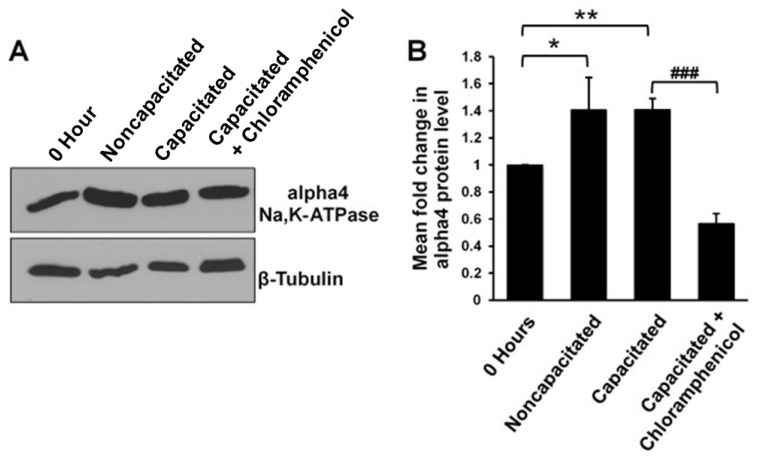
Sperm synthesize new alpha4 peptides from mitochondrial ribosomes. Sperm total protein was extracted from freshly isolated caudal sperm (0 h) or sperm incubated for 4 h in noncapacitating buffer, capacitating buffer, or capacitating buffer supplemented with chloramphenicol and were subjected to western blotting. (**A**) A representative western blot of alpha4 total protein levels. β-tubulin was included as a loading control. (**B**) Alpha4 protein levels were normalized to β-tubulin and expressed as a fold change with respect to alpha4 protein levels at 0 h. Bar chart represents the mean ± SEM fold change of three blots for three biological replicates (9 blots total). Statistical analyses were performed using a Welch’s *t*-test (* *p* < 0.05; ** *p* < 0.01) and unpaired student’s *t*-test (^###^ *p* < 0.001).

Although not quantitative, our immunofluorescent experiments suggest that the alpha4 Na,K-ATPase levels increase in the membrane of sperm over time after removal from the epididymis (compare Figure 2A to Figure 2B,C), similarly to what was reported by Jimenez et al. [24]. The authors of that study suggest that the increase in alpha4 Na,K-ATPase at the plasma membrane of capacitated sperm could be due to movement of this protein from an intracellular store to the cell surface [24]. However, it has been reported that sperm maintain the ability to translate existing mRNA to produce proteins [31,32] including alpha4 Na,K-ATPase [33]; therefore, de novo protein synthesis may underlie our observed increase in alpha4 Na,K-ATPase at the plasma membrane of capacitated sperm. To address the possibility that sperm are synthesizing alpha4 protein, we evaluated the changes in alpha4 Na,K-ATPase protein levels in sperm incubated in capacitating conditions by western blot. Total protein lysates were prepared from sperm incubated for 4 h in noncapacitating buffer, capacitating buffer, and capacitating buffer supplemented with 0.1 mg/mL chloramphenicol. Surprisingly, we observed that both noncapacitating and capacitating conditions support an increase in the total protein level of alpha4 Na,K-ATPase (Figure 5A,B). In the presence of chloramphenicol, this increase was not only abolished, but the overall level of alpha4 Na,K-ATPase decreased, suggesting that significant degradation occurs for this protein.

## 3. Discussion

The objective of this study was to investigate the expression and localization of alpha4 Na,K-ATPase throughout the development and maturation of male germ cells. We have used a highly specific anti-alpha4 antibody to show that while the majority of alpha4 Na,K-ATPase becomes expressed late in spermatogenesis, similar to the findings of Wagoner et al. [5], low levels of expression are detectible in spermatogonia and spermatocytes.

Our data also suggest that alpha4 Na,K-ATPase is found at low levels in Sertoli cells. As far as we are aware, no reports of Sertoli cells expressing the Na,K-ATPase alpha4 isoform protein have been published for any species. In addition, mouse Sertoli cell RNAseq has shown that alpha4 mRNA expression is below the levels of genes known not to be expressed in these cells (see supplementary data associated with Ref [34]). Furthermore, data from single cell RNAseq from mouse testis confirm the lack of alpha4 Na,K-ATPase mRNA in Sertoli cells [35]. These studies suggest that the alpha4 transcript is not expressed in Sertoli cells at a functional level, at least in mice. Interestingly, the alpha4 Na,K-ATPase protein that we detect in Sertoli cells is visualized as a punctate distribution with a perinuclear localization reminiscent of mature phagosomes in macrophages [36]. Considering that extensive phagocytosis is a major function of Sertoli cells [37], this perinuclear immunolabeling could be due to alpha4 protein contained within mature phagosomes taken up as part of spermatid residual bodies or apoptotic spermatogenic cells. If so, our anti-Na,K-ATPase alpha4 antiserum could serve as a useful marker to identify rat phagosomes in studies of Sertoli cell phagocytosis of natural substrates in vivo or in culture.

We have also shown that alpha4 Na,K-ATPase localization in epididymal sperm is dependent on incubation conditions. Immunofluorescent and ultrastructural localization of alpha4 in sperm incubated in capacitating conditions showed that alpha4 Na,K-ATPase was expressed not only in the sperm midpiece, but highly enriched on the plasma membrane overlying the sperm acrosome. Since we showed alpha4 Na,K-ATPase co-localized with the acrosome during spermatogenesis, we hypothesized that a small pool of alpha4 Na,K-ATPase may reside within the sperm head. While we cannot exclude this possibility, we did not visualize any gold particles within the sperm head using electron microscopy despite using permeabilizing agents and high accelerating voltages to visually penetrate through the sperm cell.

The significance of capacitation-induced alpha4 Na,K-ATPase expression along the sperm acrosome is unclear but suggests that increased Na,K-ATPase activity is involved in membrane hyperpolarization and acrosome reaction. One of the events during sperm capacitation involves the efflux of Na^+^, which is a major contributing factor to membrane hyperpolarization [38]. Interestingly, it appears that membrane hyperpolarization alone is sufficient to induce calcium influx and acrosome reaction independently of other capacitation-associated events [39]. Considering these data, the augmentation of alpha4 Na,K-ATPase along the acrosomal region of the sperm head may be involved in initiating acrosome reaction.

The activity of the Na,K-ATPase supports the activity of multiple Na^+^-dependent secondary active transporters as they use the inward-directed Na^+^ electrochemical gradient to move other solutes across the cell’s membrane. One family of these Na^+^-coupled pumps, the Na/H exchangers (NHEs) has members (NHA1, NHA2, and NHE10) that are critical for sperm motility and male fertility in mice [40,41]. Recently, a new member of the NHE family, NHE11, has been shown to localize to the sperm head adjacent to the acrosome in rats and humans [42]. It is possible that the redistribution of Na,K-ATPase alpha4 to this same region is necessary to support the activity of NHE11 to regulate the pH of the head (or the acrosome) during capacitation-related events in the sperm.

In addition to alpha4 Na,K-ATPase relocalization in sperm, we have provided evidence suggesting that the levels of detectable alpha4 Na,K-ATPase protein increases in sperm, independent of capacitation status. Over fifty years ago [43], the first evidence of protein synthesis in sperm was reported; however, this idea remains a controversial one. During spermatogenesis, sperm undergo considerable intracellular remodeling, including the loss of nearly all organelles and reconstruction of the nucleus into a highly condensed DNA-protamine complex inaccessible to the transcriptional machinery (reviewed in Refs [44,45,46,47,48]). These drastic changes have led to the widespread acceptance that sperm are transcriptionally and translationally quiescent. Even though it is unlikely that mature sperm synthesize new mRNA, existing mRNAs have been reported to be translated by chloramphenicol-sensitive ribosomes [31,32]. Mitochondria within sperm are the main source of these ribosomes, which suggests that sperm could utilize mitochondrial ribosomes for translation of nuclear-encoded mRNAs in the absence of their cytosolic counterparts.

Of the nuclear-encoded mRNAs reported to be translated by sperm, many encode proteins with established roles in capacitation and acrosome reaction [31,32]. Interestingly, alpha4 Na,K-ATPase protein levels decrease in rat sperm incubated with chloramphenicol, allowing for the interpretation that alpha4 mRNA translation may occur in mature sperm [31]. Additionally, 2D PAGE analysis of mouse sperm shows that 44 chloramphenicol-sensitive proteins are upregulated during sperm capacitation [32]. Most recently, it was shown that bovine sperm synthesize alpha4 Na,K-ATPase using mitochondrial ribosomes during capacitation [33]. While the significance of translation in sperm is not well understood, functional analysis has shown that inhibition of translation using mitochondrial ribosome inhibitors significantly reduces motility and blocks capacitation in both mouse and bovine sperm [31,32] and reduces the motility of human sperm [49].

In the present study, we have shown that the level of detectable alpha4 Na,K-ATPase increases in rat sperm in both capacitating and noncapacitating conditions, and that chloramphenicol causes a significant decrease in alpha4 protein levels. One way to interpret these results is that mitochondrial ribosomes synthesize additional Na,K-ATPase alpha4 protein using residual nuclear-encoded mRNAs in the rat sperm. However, it is unclear how this topologically complex protein (alpha4 is predicted to have 10 transmembrane alpha helices) could be synthesized and properly incorporated into the sperm plasma membrane without the presence of a fully functional endoplasmic reticulum.

If sperm are able to translate nuclear-encoded mRNAs, there are at least two possible benefits to the cell. First, translation may act as a compensatory mechanism to replace degraded proteins while the sperm reside in the female reproductive tract. Motility, the acrosome reaction and in vitro fertilization showed time-dependent deficits when mitochondrial ribosomes were inhibited with chloramphenicol [31]. This suggests that sperm are unable to maintain sperm-relevant functions without the ability to replace degrading proteins. In congruity with this idea, our results demonstrated a time-dependent decrease in alpha4 Na,K-ATPase levels in the presence of chloramphenicol.

Second, translation in sperm may act as a key maturation step to increase levels of proteins important for capacitation and acrosome reaction. At least 22 proteins which support critical sperm functions are sensitive to chloramphenicol treatment [31]. In addition, 2D-PAGE analysis shows that 74 proteins are upregulated during mouse sperm capacitation, and many of these are critical for capacitation and acrosome reaction [32]. Relevant to the current study, overexpression of the rat or human alpha4 Na,K-ATPase in transgenic mice increased both the total and progressive motility, which suggests that increased alpha4 Na,K-ATPase protein levels promote hyperactive motility [50,51].

Finally, we have provided evidence that the increase in detectable sperm Na,K-ATPase alpha4 protein occurs independently of capacitating conditions. This presents the possibility that sperm may require the ability to synthesize proteins while they reside in the epididymis. In support of this hypothesis, administering tetracycline to rats over a two-week period caused a 46% reduction in sperm count and a 69% reduction in sperm motility [52]. Since tetracycline, like chloramphenicol, only inhibits prokaryotic (or prokaryotic-like) ribosomes, this defect must stem from mitochondrial ribosome inhibition and shows that sperm viability at least partially depends on the activity of mitochondrial ribosomes. This is further supported by a study in which tetracycline-treated pseudoscorpions showed a significant decrease in sperm number and motility [53]. These sperm defects, induced in the presence of mitochondrial ribosome inhibition, demonstrate that protein production by mitochondrial ribosomes may be important and potentially required for sperm fertility.

The decrease in alpha4 Na,K-ATPase protein levels in sperm incubated with chloramphenicol may indicate that this protein is synthesized by sperm using mitochondrial ribosomes. Alternatively, this could signify that chloramphenicol prevents the translocation of the alpha4 Na,K-ATPase from an unknown intracellular compartment to the plasma membrane, or that it prevents the unmasking of the alpha4 Na,K-ATPase epitope over time in sperm incubated in vitro by undescribed mechanisms. Therefore, further experimentation is needed to understand the mechanism behind this chloramphenicol-mediated decrease in alpha4 Na,K-ATPase in sperm.

## 4. Materials and Methods

### 4.1. Reagents

All components of the sperm motility buffers (see below) were purchased from Sigma-Aldrich (St. Louis, MO, USA). The primary antibodies used in this study include an anti-alpha4 antibody generated for us by the Proteintech group using the same antigenic peptide used by Woo et al. [3] and an anti-tubulin monoclonal antibody developed by Michael Klymkowsky and obtained by us from the Developmental Studies Hybridoma Bank developed under the auspices of the NICHD and maintained by the University of Iowa, Department of Biology, Iowa City, IA 52242. Secondary antibodies include Alexa Fluor 488 goat anti-rabbit IgG, Alexa Fluor 594 goat anti-rabbit IgG, horseradish peroxidase (HRP)-conjugated goat anti-rabbit IgG, HRP-conjugated goat anti-mouse IgG, and an 18 nm gold-conjugated goat anti-rabbit IgG, all purchased from Jackson ImmunoResearch (West Grove, PA, USA). FITC-conjugated peanut agglutin (PNA; Sigma-Aldrich) was used for staining the acrosomal compartment, and Vectashield Mounting Medium with DAPI (Vector Laboratories, Newark, CA, USA) was used for staining cell nuclei.

### 4.2. Preparation of the Anti-alpha4 Na,K-ATPase Polyclonal Antibody

An alpha4 Na,K-ATPase antiserum was generated (Proteintech, Rosemont, IL, USA) by injecting rabbits with a peptide corresponding to amino acids 16–33 of rat alpha4 Na,K-ATPase [2]. Rabbits were bled, and the anti-alpha4 antibody was purified by affinity chromatography using SulfoLink columns (Thermo Scientific, Waltham, MA, USA) according to the manufacturer’s instructions, and the yield was quantified by the A280 method.

### 4.3. Sperm Isolation

Testes and sperm cells were obtained from sexually mature adult Harlan Sprague–Dawley rats following CO_2_ asphyxiation in accordance with protocols approved by Miami University’s Institutional Animal Care and Use Committee. All animal experiments were carried out in accordance with the National Institutes of Health guide for the care and use of Laboratory animals. Caudal epididymes were placed in noncapacitating modified TALPS (“mTALPS”) medium (95 mM NaCl, 4.7 mM KCl, 1.7 mM CaCl_2_, 1.2 mM KH_2_PO_4_, 1.2 mM MgSO_4_, 5.5 mM glucose, 0.27 mM pyruvic acid, 0.25 mM lactic acid, 40 mM HEPES, and 20 mM Tris, pH 7.35). Epididymes were carefully minced and incubated in mTALPS for 10 min. Motile sperm were removed from the swim up, carefully filtered with a 70 µm cell strainer, and diluted to ~1.5 × 10^7^ cells/mL and maintained at 37 °C in 5% CO_2_ for all experiments. When appropriate, sperm were incubated in capacitating mTALPS medium supplemented with 5 mg/mL BSA (Cohn Fraction V) and 25 mM sodium bicarbonate. Sperm were incubated in noncapacitating or capacitating conditions for 4 h. Sperm capacitation was qualitatively evaluated by sperm hyperactive motility observed by microscopic evaluation or quantitatively using the Hamilton Thorne TOX IVOS CASA system (Hamilton Thorne, Beverly, MA, USA). Only experiments with >70% hypermotile sperm under capacitating conditions were considered for analysis. To inhibit mitochondrial ribosomes, chloramphenicol (0.1 mg/mL) was included in capacitating mTALP when specified.

### 4.4. Immunohistochemical Analysis

Rat testes were isolated and immediately fixed in 10% neutral buffered formalin overnight at room temperature with gentle agitation. Testes were washed twice in phosphate-buffered saline (PBS), dehydrated in an ethanol series, and embedded in paraffin. The prepared tissue was sectioned at a thickness of 10 µm and attached to glass slides for staining.

For immunolocalization of alpha4 Na,K-ATPase, testis sections were deparaffinized by two exchanges of xylene and then rehydrated in an ethanol series. Antigen retrieval was performed by steaming the sections in hot citrate buffer (pH 6.0) for 30 min. The slides were cooled to room temperature and washed twice with PBS for 5 min each. To block nonspecific binding, sections were incubated with 5% normal goat serum in PBS for 1 h at room temperature. The sections were labeled with the affinity-purified anti-alpha4 Na,K-ATPase antibody (20 µg/mL) in PBS with 1% normal goat serum overnight at 4 °C. The following day, sections were washed 4 times in PBS containing 0.1% Tween20. To visualize alpha4 Na,K-ATPase, sections were labeled with an Alexa Fluor 594-conjugated goat anti-rabbit IgG diluted 1:200 in PBS with 1% normal goat serum. To aid in the interpretation of alpha4 Na,K-ATPase localization with respect to the different stages of spermatogenesis, 50 µg/mL of FITC-conjugated PNA was included to label the acrosomal compartment. After incubating at room temperature for two hours, the slides were washed 6 times in PBS with 0.1% Tween20. Slides were mounted in Vectashield with DAPI to allow for visualization of nuclei.

The fidelity of the anti-alpha4 Na,K-ATPase antibody was confirmed by preabsorbing the antibody with a 200 molar excess of the peptide used for immunization. This mixture was incubated overnight at 4 °C with gentle agitation and used the following day in place of the anti-alpha4 Na,K-ATPase antibody. To determine the specificity of the secondary antibody, an additional control experiment was performed by omitting the anti-alpha4 Na,K-ATPase antibody altogether. All fluorescent images were acquired using an Olympus FV500 Confocal Microscope (Olympus, Center Valley, PA, USA).

### 4.5. Immunocytochemical Analysis

Caudal sperm were incubated in capacitating or noncapacitating conditions and sampled at various time points. The sperm were pelleted by centrifugation (700 g × 5 min), gently resuspended in 4% paraformaldehyde and 0.25% glutaraldehyde in PBS, and fixed for 30 min at room temperature. The fixed sperm were washed once with PBS, smeared onto slides, and air-dried. Sperm were then washed with PBS, blocked, and immuno-labeled as described above, except that Alexa Fluor 488-conjugated goat anti-rabbit IgG was used as a secondary. Sperm nuclei were counterstained with DAPI.

### 4.6. Immunogold Scanning Electron Microscopy

Sperm were prepared for immunogold staining using the similar methods described above. In brief, sperm were collected by centrifugation, washed with PBS, fixed, and incubated on poly-L-lysine-coated coverslips to allow them to adhere. The sperm were then blocked, incubated with the anti-alpha4 Na,K-ATPase antibody, washed, and labeled with an 18 nm gold-conjugated goat anti-rabbit IgG used at a dilution of 1:10. To help preserve antibody–antigen interactions, the samples were postfixed in 2.5% glutaraldehyde in PBS for 30 min at room temperature after washing off unbound secondary antibody. Following several washes with PBS, the coverslips were rinsed briefly in distilled water and serially dehydrated in ethanol in concentrations from 30% to 100%. The samples were then dried using a Tousimis Samdri-780A Critical Point Dryer (Tousimis, Rockville, Maryland, USA) following the manufacturer’s instructions. Finally, the samples were carbon-coated using a Denton 502A Vacuum Evaporator (Denton Vacuum, Moorestown, NJ, USA). Samples were imaged using the Zeiss Supra 35 VP FEG SEM (Zeiss, White Plains, NY, USA using a 12kV accelerating voltage, 7 mm working distance, and 30 µm NA.

### 4.7. Immunoblotting

To prepare homogenates for the multi-tissue western blot, the testis, kidney, brain, skeletal muscle, heart, liver, and lung were removed from euthanized adult rats and snap-frozen in liquid nitrogen. Tissues were then thawed in RIPA buffer (Santa Cruz Biotech, Dallas, TX, USA) supplemented with Halt protease inhibitor (Pierce-Thermo Fischer, Waltham, MA, USA) and homogenized. The homogenate was centrifuged at 20,000× *g* for 30 min at 4 °C, and the supernatant was transferred to a new tube for quantification by BCA assay (Pierce). To prepare sperm lysate, aliquots were taken from the various time points in the above experiments, pelleted at 700 g for 10 min, and resuspended in RIPA buffer supplemented with protease inhibitor. The sperm were sonicated for 30 s and incubated on ice for 30 min with occasional agitation. The cell lysates were then centrifuged at 14,000× *g* for 15 min at 4 °C to pellet cell debris.

Approximately 10 µg of protein from each tissue or 3 × 10^7^ sperm were incubated in Laemmli Sample Buffer (BioRad, Hercules, CA, USA) for 1 h at 37 °C and electrophoretically separated by SDS-PAGE. Following transfer onto PVDF membranes, the blots were washed with TBS (pH 7.5) and blocked with 5% NFDM in TBS for 1 h at room temperature. The blots were then incubated overnight at 4 °C with 1 µg/mL affinity-purified anti-alpha4 Na,K-ATPase or anti-tubulin diluted 1:1000 in TBS with 5% non-fat dry milk (NFDM). The following day, the blots were washed three times with TBS containing 0.05% Tween20, then incubated with HRP-conjugated anti-mouse or anti-rabbit secondary antibodies diluted 1:10,000 in TBS containing 5% NFDM for 1 h at room temperature. Unbound antibody was removed with 6 washes of TBS with 0.05% Tween20. Lastly, bands were visualized by incubating blots with Supersignal West Pico Substrate (Thermo Scientific) and developed with autoradiographic film. The bands were quantified using ImageJ software (version 2.0) and alpha4 Na,K-ATPase bands were normalized to the β-tubulin loading control. Each sample was run in triplicate and the experiment was repeated with three different animals (9 blots total).

## 5. Conclusions

In summary, our testes immunofluorescence studies show that localization of the alpha4 Na,K-ATPase shifts during multiple stages of sperm maturation. Our immunofluorescence and immune-SEM experiments showed that alpha4 Na,K-ATPase becomes detectable along the sperm head on the plasma membrane overlying the acrosome. Because this translocation of alpha4 Na,K-ATPase to the sperm head was dependent on the presence of capacitating stimuli (bicarbonate, cholesterol acceptor/BSA), the localization of alpha4 may be a useful biomarker for sperm capacitation (at least in rats) and implicates this testis-specific protein as a potential regulator of acrosome reaction. One caveat of our study was the absence of markers of capacitated and noncapacitated sperm, and, therefore, we cannot rule out that some of these changes simply occur under capacitating conditions and not necessarily in capacitated sperm. Considering the importance for the Na,K-ATPase alpha4 for motility in rodent and human sperm and fertility in mice [7,9,11], it is not surprising that attempts to develop Na,K-ATPase alpha4 inhibitors for use as male-specific contraceptive agents are underway [54,55]. Caution should be taken in view of the recent description of a mutation in ATP1A4 in a family with familial hemiplegic migraine (FHM) [56], suggesting that Na,K-ATPase alpha4 is expressed in the human brain, and, therefore, inhibitors that are able to cross the blood–brain barrier may have significant negative side-effects.

## Data Availability

The data supporting the findings of this study are available within this article.
